# Attitudes, Knowledge and Clinical Practice of Health Professionals towards Psychological Disorders in Cancer Patients: An Observational Study

**DOI:** 10.3390/diseases12070141

**Published:** 2024-07-01

**Authors:** Maria Chiara Carriero, Antonio Leo, Alessia Lezzi, Roberto Lupo, Luana Conte, Annarita Fanizzi, Raffaella Massafra, Elsa Vitale, Antonio Carriero

**Affiliations:** 1 Independent Researcher, 20100 Milano, Italy; 2 “Santa Chiara” Institute, Via Campania, 1, 73100 Lecce, Italy; 3 ANT Italia ONLUS Foundation (National Cancer Association), 73100 Lecce, Italy; 4 “San Giuseppe da Copertino” Hospital, ASL (Local Health Authority), 73100 Lecce, Italy; 5 Laboratory of Biomedical Physics and Environment, Department of Mathematics and Physics “E. De Giorgi”, University of Salento, Via Antonio Miglietta, 5, 73100 Lecce, Italy; 6 Laboratory of Interdisciplinary Research Applied to Medicine (DReAM), University of Salento and ASL (Local Health Authority), Via Antonio Miglietta, 5, 73100 Lecce, Italy; 7 Laboratorio di Biostatistica e Bioinformatica, IRCCS Istituto Tumori “Giovanni Paolo II”, Viale Orazio Flacco 65, 70124 Bari, Italy; 8 Scientific Directorate, IRCCS Istituto Tumori “Giovanni Paolo II”, Viale Orazio Flacco 65, 70124 Bari, Italy; 9 Department of Oncology, “V. Fazzi” Hospital, Piazza Filippo Muratore, 1, 73100 Lecce, Italy

**Keywords:** depression, demoralization, oncology, psychology, mental health

## Abstract

Background: The suffering associated with a cancer diagnosis can find different channels to express itself: sleep disorders, psychiatric disorders, sexuality. These are not always analyzed by health professionals, but they have an impact on the patient’s quality of life and on the outcome of the disease. Methods: An observational study was conducted in order to investigate attitudes, knowledge and clinical practice towards psychological symptoms in cancer patients. Results: A total of 132 clinicians from all Italian regions responded. In total, 99.2% (n = 131) considered the figure of the psychologist useful in the oncology field and recommended him/her in clinical practice (n = 115; 87.7%), especially in the terminal phase of the illness (58.6%; n = 99). Despite the importance given to the figure of the psychologist, psychiatric disorders are not diagnosed. Only 20.0% (n = 26) identified depressive disorder as accurate and only 33.9% (n = 43) identified demoralization syndrome as accurate. Conclusions: Results prove the need for training on psychological disorders in oncology and the emotional repercussions of cancer illness.

## 1. Introduction

Cancer is a disease that has a strong impact on the patient’s quality of life. In addition to the symptoms of the disease and the effects of treatment, the patient is also faced with questions about the meaning of life, suffering and death. The suffering associated with a cancer diagnosis can find different channels to express itself: somatic symptoms, sleep disturbances, lack of appetite and psychiatric disorders, which are not always analyzed by physicians [1]. Depression, demoralization and despair often occur in cancer patients and these have a significant impact on the patient’s quality of life and the outcome of the disease [2,3]. These clinical conditions have specific clinical signs, symptoms and criteria [4,5].

A diagnosis of depression has as its main symptoms depressed mood and loss of interest or pleasure in one’s activities [5,6]. Patients with major depression complain of persistent sadness, a generalized sense of feeling bad and a sense of hopelessness or despair. Typically, patients describe a sense of guilt and a feeling of having failed their loved ones. They complain of decreased energy, easy distraction, poor concentration and insufficient sleep. Another important feature is the anhedonia, because patients are unable to feel pleasure. In addition, major depression tends to be recurrent and may occur in multiple episodes throughout the patient’s life [4]. Although it is very common in cancer patients, with prevalence rates of 14.3% for the diagnosis of Major Depressive Episode and 9.6% for Minor Depressive Episode [7,8], with a range of 17–25% in the elderly [5,6,7,8,9], depression is difficult to diagnose in cancer patients [10]. It is becoming an increasingly urgent problem because it is associated with decreased quality of life, more rapid progression of cancer symptoms, and more metastases and pain [5,6,7,8,9,10,11].

In light of this, most cancer patients with psychological distress do not see mental health professionals, but rather their oncologists, who often have little knowledge of assessing psychological disorders [12], are unable to detect psychiatric disorders [13] due to lack of confidence in assessing with psychometric instruments [14] and do not have the time to address psychological problems [12].

Therefore, in clinical practice, the lack of psychometric instruments may lead to an underestimation of demoralization, sometimes interpreted as a “normal state of sadness” [15]. In addition, it is worth considering the diagnosis of depression in elderly patients with cancer. In this case, cancer and depression symptoms might have distinct but overlapping profiles (e.g., fatigue, lethargy, suicidal ideation, pain, anxiety, or difficulty adapting to the cancer diagnosis), so specific testing might be useful [5].

Psychiatric and psychological assessment must be a key component in the management of patients with cancer. Therefore, it is essential that psychological disorders are recognized at an early stage, so that resources can be offered by a professional and psychological distress can be reduced [2]. In addition, if physicians were trained in recognizing psychiatric disorders, they could increase their accuracy in treating the patient. Based on this, in our study, we investigated the level of knowledge, clinicians’ attitudes and practice toward symptoms of depression and demoralization in cancer patients. Few studies have been conducted in this regard, and the literature reflects the consequent need for the study of this issue. The aim of this study is to investigate healthcare providers’ attitudes, knowledge and practice regarding psychological disorders and symptoms in cancer patients. We also investigate the experience and attitudes of clinicians towards the discipline of Psychology in oncology.

## 2. Materials and Methods

### 2.1. Design

An observational, cross-sectional, multicenter study was conducted between January 2019 and January 2020.

### 2.2. Sample and Data Collection Procedures

Participants were recruited by various methods, including face-to-face and online dissemination of a questionnaire sent by the study leader to directors of oncology departments in Italy. Approximately 100 e-mails were sent to each department director, containing a brief presentation of the survey and the link to access the online questionnaire. Some decided to participate in the study; from others, we did not receive a response and some did not want to participate. Moreover, the questionnaire was sent through the most common social platforms (WhatsApp, Facebook), as the purpose required reaching a large number of participants. This allowed participants to answer the questions directly from their devices. Each participant who voluntary agreed to participate in this study completed an on-line web form. A total of 132 healthcare providers, including physicians and nurses, agreed to participate in the study.

Regarding recruitment criteria, we decided to include healthcare workers, all nurses and physicians, regardless of health department, in the study. Healthcare workers could work in public or private Italian facilities or practices, with a minimum age of 23 years and maximum age of 65 years and with at least 12 months of work experience. The study was conducted on the sample described in order to conduct an exploratory investigation, in a field still little explored by the literature in the oncology field, unlike other fields, such as dementia [16]. A sample-size study was not performed, and equal numbers of nurses and physicians were not selected because we spread the questionnaire widely and the survey was exploratory. Healthcare workers who responded to the invitation for online completion and who agreed to complete the questionnaire were recruited into the study. Healthcare professionals who did not give consent were excluded.

### 2.3. The Questionnaire and Data Collection Tools

To enable the collection of data and to analyze the “knowledge, perceptions of local services and attitudes of health professionals toward the discipline of Psychology in oncology,” a questionnaire was created ad hoc and without any previous validation studies. All sections of the questionnaire were computerized through the use of a present form from the Google Drive platform. We decided to divide the questionnaire into three sections and with items structured in multiple-choice questions.

The first section contained general sociodemographic information (Table 1). The second section contained questions regarding experience with the discipline of Psychology in oncology (Table 2). The third section asked questions to investigate knowledge of some psychological disorders that may develop in oncology (Table 3).

The variables analyzed in the section entitled “General socio-demographic information” were gender, type and year of degree, specialization, job and other academic qualifications. 

In the section entitled “Experience with the psychological discipline in oncology,” we analyze the views of health professionals on the figure of the psychologist in oncology, the experiences of emotional management of patients with cancer and the perception of health services in Italy.

Among the various psychological disorders that can occur in oncology, we decided to analyze the knowledge of health professionals on depressive disorder, demoralization disorder and despair. The section entitled “Knowledge of some psychological disorders that can develop in oncology” uses a multiple-choice scale, with only one correct answer. The correct answer is the response that shows the DSM-5 diagnostic criteria. If no opinion was expressed, it was assumed that the participant did not know the answer.

All variables were considered as categorical variables and assessed as frequencies and percentages, including missing given answers. Next, chi-squared tests were performed on psychological attitudes according to training sources declared by participants. All tests were performed using a significance level of 0.05. All variables were analyzed using R-Studio version 3.6.1 software.

### 2.4. Ethical Considerations

Data were collected while respecting the confidentiality and anonymity of the participants. Within the questionnaire presentation, the ethical characteristics of the study were stated. It was emphasized that participation was voluntary, and that the participant could decline to participate in the protocol whenever desired. Those interested in participating were given an informed consent form, which reminded them of the voluntary nature of participation, as well as the confidentiality and anonymous nature of the information. Questionnaires were administered only to healthcare professionals who agreed to participate in the survey by signing informed consent. The study design was explained and presented to the heads of each Operating Unit; only after the participants’ consent was given was the survey initiated. Since this was an online questionnaire, no consent was requested from the Ethics Committee. In addition, to ensure that the questionnaires were anonymous and to allow identification of participants, a sequential identification (ID) number was assigned to each registered participant. Each questionnaire, therefore, had an ID number that corresponded to the database ID.

## 3. Results

### 3.1. Demographic Characteristics of the Sample

A total of 132 clinicians across the Italian regions responded. Overall, 59.8 (n = 79) were female, 62.1% (n = 82) were from Southern Italy, 53% (n = 70) had a degree in Medicine and Surgery and 34 (25.7%) had a degree in Nursing (Table 1).

In total, 99.2% (n = 131) consider the figure of the psychologist useful in the oncological field, so much so as to recommend him/her in clinical practice (n = 115; 87.7%), especially in the terminal phase of the disease (47% n = 62). Furthermore, 75.2% (n = 97) of the sample believe that training courses on aspects of psychology in oncology are useful. In addition, 86.4% (n = 116) of the sample sought the advice of a psychologist in the emotional management of a patient (Table 2). Regarding the knowledge of psychological disorders, it emerged that 20.0% (n = 26) of the sample identify depressive disorder as “an emotional experience connoted by depressed mood, loss of interest, weight loss, insomnia/hypersomnia, psycho-motor agitation, feelings of self-evaluation, suicidal ideation and decreased attention span,” while 33.9% (n = 43) identified demoralization syndrome as “an emotional experience characterized by social isolation, loss of hope, feelings of entrapment, and a desire to give up”. A total of 56.1% of the sample (n = 69) reported assessing symptoms through the clinical interview. Regarding the knowledge of psychological disorders, it emerged that 20.0% (n = 26) of the sample identify depressive disorder as “an emotional experience connoted by depressed mood, loss of interest, weight loss, insomnia/hypersomnia, psycho-motor agitation, feelings of self-evaluation, suicidal ideation and decreased attention span,” while 33.9% (n = 43) identified demoralization syndrome as “an emotional experience characterized by social isolation, loss of hope, feelings of entrapment, and a desire to give up.” Furthermore, 56.1% of the sample (n = 69) reported assessing symptoms through the clinical interview.

### 3.2. Survey on “Knowledge of Some Psychological Disorders That May Develop in Oncology” According to Psychological Training Sources Declared

As shown in Table 3, most of the participants who declared that they did not have any psychological training, or that they had undertaken basic training thanks to conference attendance, declared that they recommended a psychologist to their patients (*p* = 0.006). Additionally, all the participants agreed to consider training in psychological matters to take care of their oncology patients. Training could include several typologies, without any specific significant difference (*p* = 0.708). However, the interviewers did not consider the psychologist consultation as the most important aspect in emotional management (*p* = 0.124).

More interesting were the answers given for item no. 18, as most of the participants answered that they believed that healthcare systems in the south of Italy were lacking in efficiency and timeless. However, most of the participants also considered prejudice towards the southern healthcare system. These considerations were affirmed by both the participants who took conferences and those who did not (*p* < 0.001). Finally, for the last items, there was no statistical significance in the participants’ beliefs.

## 4. Discussion

The primary purpose of this survey was to investigate physicians’ attitudes, knowledge and practice regarding psychological symptoms in cancer patients. We investigated physicians’ and nurses’ knowledge about some psychological disorders because the first goal of this study was to identify whether there is a need for training and education on the identification of psychological symptoms and disorders in cancer patients, to lead to improved interventions and diagnosis formulation. For example, whether the participants considered the figure of the psychologist important, whether they had recommended a psychologist to their patients, whether they had prescribed antidepressant or neuroleptic drugs to patients, etc. This was also investigated to determine the relationship between clinical practice and clinicians’ attitudes. In investigating perceptions of oncology services in Italy and the experience of health professionals in the Psychology discipline during their clinical practice, it was seen that 99.2% (n = 131) of the professionals who participated in the survey consider the figure of the psychologist useful in oncology. But all the participants agreed to consider training in psychological matters to take care of their oncology patients. Training could include several typologies, without any specific significant differences (*p* = 0.708).

These findings are borne out by the fact that in the past decade, the training of health professionals has also included increased attention to patients’ psychosocial problems, opening up new opportunities for psychologists in collaborating with medical services and structuring integrated intervention plans [17].

The results of our study show how important the contribution of the psychologist is: a large proportion of the sample, 87.7% (n = 114), sought the advice of a psychologist during their work experience for the effective emotional management of patients (Table 2). However, other respondents did not consider the psychologist consultation the most important factor in emotional management (*p* = 0.124).

There is, however, according to Carretti and La Barbera [18], a specific problem that stems from the physician’s difficulty in noticing and taking into account personal characteristics as a risk factor for somatic disorders or diseases [18]. In fact, despite the importance given to the figure of the psychologist, psychiatric disorders are often not diagnosed by health professionals [13]. A delayed and inadequate response to the patient’s psychological problem leads to the persistence and worsening of symptoms, increasing the person’s discomfort and resulting in greater expense for the health service [19].

In a study by Freeling et al. [20], general practitioners failed to recognize depression in the presence of an organic illness and did not refer their patients to specific specialists. Although general practitioners recognize and manage a large number of physical illnesses, knowledge of mental disorders is therefore neglected. Jorm et al. [21] introduced the term ‘mental health literacy’ and defined it as “the knowledge and beliefs about mental disorders that aid in recognition, management, and prevention.” Mental health literacy consists of the ability to recognize psychological distress, attitudes that facilitate recognition, knowledge of risk factors, interventions and seeking appropriate mental health information [22]. The importance of health literacy for physical health is recognized; the area of literacy for mental health, on the other hand, is neglected [22]. Only 20.0% of our sample (n = 26) identified depressive disorder accurately, that is, as “an emotional experience marked by depressed mood, loss of interest, weight loss, insomnia/hypersomnia, psycho-motor agitation, feelings of self-evaluation, suicidal ideation, and decreased attention span.” These results agree with WHO data reporting that a good 50% of depression cases go undiagnosed because many treating physicians are unable to recognize it, even though the disorders it produces make it more disabling than other diseases, such as osteoarthritis, hypertension and diabetes [23]. Only 33.9% of the sample (n = 43) identified demoralization syndrome as “an emotional experience characterized by social isolation, loss of hope, a sense of entrapment, and a desire to give up.” These findings demonstrate the need for education regarding psychological disorders that may occur in the oncology setting. Patients with comorbid mental health and substance use disorders are at greater risk for mortality and have higher cancer care costs [24]. A systematic literature review with 17 studies and more than 280,000 patients found that people with depression and/or anxiety with breast cancer had a higher rap-port of risk in terms of recurrence, cancer-specific mortality, and all-cause mortality [25]. Given that a person with cancer may be at increased risk of suicide and the development of psychiatric disorders—most notably, depressive and anxiety disorders—Goldstone underscored the importance of identifying patients who are in need or in potential need of psychiatric treatment [24]. According to Goldstone [24], many providers are uncertain about what to do if a patient reports suicidal thoughts, and in many cases, there is not a clear procedure to follow in the institution or the organization itself. In addition, psychiatric evaluation should be a key component in the management of patients with cancer, and in this regard, only 56.1% of the sample (n = 69) reported that they assess psychological symptoms through the clinical interview and not through specific psychological assessment tests. Screening for psychological/psychiatric distress should be conducted at baseline and at regular intervals. If a provider has identified a patient with symptoms that may indicate some type of mental health disorder, the question then becomes whether to treat the patient or to refer them to a psychiatric specialist. Goldstone has created an algorithm to help oncology providers decide whether to treat themselves or refer [24]. According to Goldstone, it is important not only to perform baseline screening, but also to undertake ongoing screening. Knowing whether symptoms of depression and anxiety are medication-related is critical as the risk–benefit of continuing to take a medication must be weighed against adding another medication to treat symptoms [24]. To help distinguish between the symptoms of depression and demoralization and the side effects of the disease, it may be useful instead to present standardized psychological assessment tools [15]. The results demonstrate the need for training in psycho-oncology and the impact and emotional repercussions of cancer illness [26,27]. Psycho-oncology is a branch of psychology that is oriented to a patient whose distress depends on the traumatizing situation of the disease, and it is also oriented to improve the quality of communication between doctors, patients and their families. This is because adaptation to the disease and treatments also depends on the relational approach of the treating team. In addition to investigating the knowledge of some psychological disorders that can develop in oncology, there is another interesting finding. Most of the participants in our study responded that they believed that healthcare systems in southern Italy are more deficient in terms of efficiency. However, most of the participants also considered this a bias against the southern healthcare system. These considerations were affirmed by both the participants who had attended conferences and those who had not (*p* < 0.001). All the rankings agree that in Italy, there is a perception of higher-quality health services in the northern and central regions and of lower quality in the south. A recent study by C.R.E.A. Health [28] provides a multidimensional assessment of the performance of individual regional health systems. It would appear that the northern and central regions are all at the top of the ranking, while the southern regions are at the bottom. Thus, the gap between the northern and southern regions in healthcare is still excessive [29]. Citizens, of course, are also aware of this. Therefore, it is not surprising that residents in southern regions go elsewhere for treatment. This is the well-known phenomenon of health mobility: every year, about half a million patients are admitted in a region other than their region of residence. Therefore, it is time to think about a health-recovery plan, with the need to popularize the urgency of a holistic approach to health, and also to an overall improvement in the quality of all health services [30,31,32].

### Limits

The interpretation of the results of this study must take into account some limitations, including the choice of the electronic dissemination of the questionnaire, which may have excluded nursing staff and physicians who might have been familiar with the diagnoses covered in the study, which could have led to possible bias selection. The limitation of the study is the presence of some missing data that could have affected the final results, although the missing answers were not found in items uniquely determining the purpose of the study. Finally, the choice of electronic disclosure for the questionnaire may have excluded physicians and nurse practitioners with a low level of IT skills. Furthermore, the data were collected online, and there was no iteration with the participants working in oncology departments. Moreover, although this is a large and heterogeneous sample and distributed over the nation, it is not possible to generalize the data to the entire category. The sample included more nurses than physicians. Therefore, the sample is not representative of the entire community of nurses and physicians. Another limitation is the consideration of physicians and nurses from all departments, not oncology departments exclusively. Additionally, the questionnaire administered was created ad hoc, without conducting a validation study beforehand. However, this could be considered as a preliminary study that needs to be deepened with other studies, which nevertheless helps to investigate the chosen topic in the meantime.

## 5. Conclusions

Identifying mental disorders in patients with cancer presents a challenge to clinicians. However, if healthcare providers were trained in recognizing psychiatric disorders, they could increase their accuracy in treating cancer patients. This lack of knowledge about mental health may place a limitation on the implementation of care and may deny adequate support to patients [22].

Patients must be helped to cope with the possibility of having a mental disorder with physical illness. However, despite these difficulties, research and the Diagnostic and Statistical Manual of Mental Disorders (DSM-5) diagnostic criteria provide guidance in identifying psychological disorders.

## Figures and Tables

**Table 1:** Socio-demographic characteristics (n = 132).

Sampling Characteristics	n (%)
Gender	125
Male	46 (34.8)
Female	79 (59.8)
Missing	7 (5.3)
Healthcare profession	
Physicians	58 (44)
Registered nurses	23 (17)
Other healthcare professions	51 (39)
Healthcare education	
Biology degree	2 (1.5)
Laboratory Technician degree	2 (1.5)
Medicine and Surgery degree	34 (25.8)
Physical/Rehabilitation therapy degree	1 (0.8)
Nursing degree	70 (53)
University degree	22 (17)
Psychology degree	3 (2.2)
Other healthcare specialization	2 (1.5)
Geographical area of residence	132
Northern Italy	3 (2.3)
Central Italy	47 (35.6)
Southern Italy	82 (62.1)
Education	132
Degree in Medicine and Surgery	70 (53)
Degree in Biology	2 (1.5)
Degree in Nursing	34 (25.7)
Degree in Physics	1 (0.8)
Degree in Psychology	1 (0.8)
Degree in TSRM (medical radiology technician)	14 (10.6)
Other	10 (7.6)

**Table 2:** Experience with the Psychology discipline in oncology.

Experience with the Psychology Discipline in Oncology	n (%)
Item no. 1: What is a depressive disorder?	132
An emotional experience characterized by sadness, distress and despair due to bereavement	4 (3)
Feelings of hopelessness, personal failure to cope with a stressful situation, lack of motivation to act, loss of life purpose	17 (12.9)
An emotional experience characterized by symptoms involving cognitive, neurovegetative, psychomotor and mood tone	17 (12.9)
An emotional experience characterized by depressed mood, loss of interest, weight loss, insomnia/hypersomnia, psychomotor agitation, feelings of self-evaluation, suicidal ideation, decreased attention span	26 (19.7)
All of the above	70 (53)
Missing	8 (6)
Item no. 2: What is demoralization syndrome?	132
An emotional experience characterized by episodes of depressed mood, accompanied by low self-esteem and loss of interest or pleasure in activities	20 (15.1)
An emotional experience characterized by social isolation, loss of hope, a sense of entrapment, and a desire to give up	42 (32.6)
State of hopelessness and depression	21 (15.9)
Engaging in behavior against common morality after learning of the cancer prognosis	4 (3)
All the above	45 (34)
Item no. 3: What is despair?	132
Does not exist as a nosographic entity	12 (9)
Synonym for demoralization	7 (5.3)
State of mind resulting from loss of hope and characterized by a sense of discouragement	93 (70.4)
The patient’s sense of freezing when faced with the communication of the diagnosis	11 (8.3)
The set of neurovegetative and cognitive reactions in relation to failure to respond to therapy	7 (5.3)
Missing	2 (1.5)
Item no. 4: Do you think psychologists are useful in oncology?	132
Yes	131 (99.2)
No	1 (0.8)
Item no. 5: Have you recommended a psychologist to your patients in your clinical practice?	132
Yes	115 (87.1)
No	16 (12.1)
Missing	1 (0.8)
Item no. 6: If yes, at what stage of the disease	132
Pre-surgical	57 (33.7)
During chemo/radiotherapy treatment	10 (5.9)
Terminal phase	62 (47)
All of the above	3 (1.8)
Item no. 7: How often do you administer antidepressant and/or neuroleptic medications?	132
Never	26 (19.7)
Almost never	27 (20.4)
Sometimes	48 (36.4)
Quite often	20 (15.2)
Very often	8 (6)
	3 (2.2)
Item no. 8: Do you think professional development and training courses on aspects of cancer psychology are helpful?	132
Yes	128 (96.2)
No	4 (3)
Missing	1 (0.8)
Item no. 9: Have you ever had to recommend an interview with a psychologist?	132
Yes	116 (86.4)
No	16 (12.1)
Missing	2 (1.5)
Item no. 10: If yes, how often?	132
Daily	12 (9)
Weekly	30 (22.7)
Several weeks	17 (12.9)
Monthly	35 (26.5)
Other	13 (9.8)
Missing	25 (19)
Item no. 11: Have you ever consulted a psychologist?	132
Yes	97 (75.2)
No	29 (22.5)
I already have psychological knowledge	3 (2.3)
Missing	3 (2.27)
Item no. 12: If you already have knowledge in the field of Psychology, can you indicate how it was developed?	132
Conferences/courses	44 (33.3)
Masters	6 (4.5)
Field experience	43 (32.6)
Other	12 (9)
Missing	27 (20.4)
Item no. 13: “Patients with cancer often have symptoms of distress.” How do you assess these symptoms?	132
With the Distress Thermometer	6 (4.5)
Using other scales	14 (10.6)
By clinical interview	69 (52.2)
I do not evaluate	34 (25.7)
Missing	9 (6.8)
Item no.14: “More and more Italians are going for treatment in regions other than their regions of residence.” These statistics show a significant migration flow for health reasons from south to north. Why?	132
Healthcare offerings often lacking, in terms of both efficiency and timeliness	57 (43.18)
Patient has knowledge, physicians and/or relatives	18 (13.6)
Patient prejudice against hospitals in the south	44 (33.3)
Unsatisfactory communication and relationship with physicians and nursing staff	13 (9.8)
Item no. 15: Have you ever had a patient who had difficulty starting treatment? Why?	132
Difficulties in planning participation in treatment and passive patient attitudes toward obligations or duties	20 (15)
Psychological dynamics and maladaptive strategies that negatively affect treatment	49 (37.12)
Pessimism about treatment and its benefits	30 (22.7)
Difficulty tolerating treatment side effects	16 (12.1)
No, it has never happened	17 (12.9)
Item no. 16: Have you ever had a patient, already in treatment, who had difficulty with treatment? Why?	132
Difficulties in planning participation in treatment and patient’s passive attitude toward obligations or duties	14 (10.6)
Psychological dynamics and maladaptive strategies that negatively affect treatment	32 (24.24)
Pessimism about the treatment and its benefits	25 (18.9)
Difficulty tolerating treatment side effects	47 (35.6)
No, this has never happened	12 (9)

**Table 3:** Knowledge of some psychological disorders that may develop in oncology based on the learning source.

Items Proposed/Learning Source	Conferences and Courses n (%)	Masters n (%)	Field Experience n (%)	Other n (%)	No Training (%)	*p*-Value
Item no. 17: In your clinical practice, do you happen to recommend the psychologist to your patient(s)?						
Yes	38 (28.8)	4 (3)	28 (21.2)	7 (5.3)	38 (28.8)	0.006 *
No	6 (4.5)	0 (0)	1 (0.8)	1 (0.8)	9 (6.8)	
Item no. 18: Do you think it would be useful to hold training and refresher courses in which issues related to aspects of cancer psychology are addressed in addition to medical aspects?						
Yes	44 (33.3)	4 (3)	28 (21.2)	8 (6.1)	43 (32.6)	0.708
No	0 (0)	0 (0)	1 (0.8)	0 (0)	3 (2.3)	
Item no. 19: Have you ever had to deal with the emotional management of a cancer patient and found it helpful to recommend an interview with a psychologist?						
Yes	39 (29.5)	4 (3)	28 (21.2)	8 (6.1)	35 (26.5)	0.211
No	5 (3.8)	0 (0)	1 (0.8)	0 (0)	12 (9.1)	
Item no. 20: Have you ever had to deal with the emotional management of a patient and found it helpful to consult with a psychologist?						
Yes	34 (25.8)	4 (3)	25 (18.9)	8 (6.1)	32 (24.2)	0.124
No	10 (7.6)	0 (0)	4 (3)	0 (0)	15 (11.4)	
Item no. 21: “The number of Italians who go for treatment in regions other than their region of residence is increasing.” “In particular, these statistics show a significant migration flow for health reasons from the south to the north.” What do you think this is due to?						
Healthcare offerings often lacking in both efficiency and timeliness	27 (20.5)	0 (0)	16 (12.1)	3 (2.3)	28 (21.2)	>0.001 *
Patient has acquaintances, physicians and/or relatives elsewhere	4 (3)	4 (3)	4 (3)	3 (2.3)	1 (0.8)	
Patient prejudices about southern hospitals	10 (7.6)	0 (0)	0 (0)	1 (0.8)	15 (11.4)	
Unsatisfactory communication and relationship with physician and nursing staff	3 (2.3)	0 (0)	0 (0)	1 (0.8)	3 (2.3)	
Item no. 22: Has it ever happened to you that a patient who has received a diagnosis, but is not yet in treatment, shows difficulties in undergoing the same? What do you think this is due to?						
Difficulty in planning participation in treatment and passive attitude of the patient toward obligations or duties	7 (5.3)	0 (0)	7 (5.3)	1 (0.8)	5 (3.8)	0.328
Psychological dynamics and maladaptive strategies that negatively affect treatment	17 (12.9)	3 (2.3)	13 (9.8)	4 (3)	20 (15.2)	
Pessimism about the treatment and its benefits	10 (7.6)	1 (0.8)	6 (4.5)	1 (0.8)	8 (6.1)	
Difficulty tolerating treatment side effects	6 (4.5)	0 (0)	2 (1.5)	2 (1.5)	2 (15)	
Never happened	4 (3)	0 (0)	1 (0.8)	0 (0)	0 (0)	

* *p* < 0.05 is statistically significant.

## Data Availability

Data are available upon reasonable request from the first author.
